# Supplementary material for: Effectiveness of Lyoprotectants in Protein Stabilization During Lyophilization
Source: Pharmaceutics. 2024 Oct 21;16(10):1346. doi: 10.3390/pharmaceutics16101346 (PMC11510631; doi:10.3390/pharmaceutics16101346)
Supplement: Supplementary file 1 [file pharmaceutics-16-01346-s001.zip › pharmaceutics-3189083-supplementary.pdf]

Supplementary Table S1: Search Strategy and Prisma Checklist

Title	Year	DOI	Reviewer 1 (Vinoothini)	Reviewer 2 (Nur Hazirah)	Final decision: include or excluded
Deep muscle-proteomic analysis of freeze-dried human muscle biopsies reveals fiber type-specific adaptations to exercise training	2021	10.1038/s41467-020-20556-8.	-Research focuses on biological adaptations in muscle fibers, not on the preservation techniques and no exploration of lyoprotectant	The research involves freeze-drying human muscle biopsies, but the freeze-drying here is simply a sample preservation technique. The study does not analyze how lyoprotectants function or contribute to stabilization in these samples.	Excluded
Strawberries Improve Pain and Inflammation in Obese Adults with Radiographic Evidence of Knee Osteoarthritis	2017	10.3390/nu9090949	study uses freeze-dried strawberry powder, it does not delve into the mechanisms or formulations of lyoprotectants used in the freeze-drying process. It does not explore how proteins or other bioactive compounds are stabilized during lyophilization	No lyoprotectant formulation was found.	Excluded
Nebulised surfactant for the treatment of severe COVID-19 in adults (COV-Surf): A structured summary of a study protocol for a randomized controlled trial	2020	https://doi.org/10.1186/s13063-020-04944-5	Not relevent to topic : no lyoprptectant was found in the study	The paper does not address the technical aspects of freeze-drying (lyophilization) or the role of lyoprotectants in stabilizing proteins during this process.	Excluded
Safety evaluation of a lyophilized platelet-derived hemostatic product	2018	10.1111/trf.14972	The study does not explore the use of lyoprotectants or the mechanisms by which they stabilize proteins during lyophilization. While the product is lyophilized, the research is concerned with its medical application rather than the chemical or biological processes involved in preserving protein structure	Not relevent to topic	Excluded
A new fully liquid presentation of MenACWY-CRM conjugate vaccine: Results from a multicentre,	2021	10.1016/j.vaccine.2021.09.068	vaccine is presented in a liquid form, the study does not involve lyophilization (freeze-drying) or the use of lyoprotectants to preserve the vaccine.	The role of lyoprotectants, which are critical in protecting proteins during the freeze-drying process, is not discussed in the paper.	Excluded

randomised, controlled, observer-blind study.					
Freeze-dried bilberry (Vaccinium myrtillus) dietary supplement improves walking distance and lipids after myocardial infarction: an open-label randomized clinical trial.	2019	10.1016/j.nutres.2018.11.008	No Discussion on Protein Stabilization or Lyoprotectants	Although freeze-drying is used to prepare the bilberry supplement, the study does not investigate the use of lyoprotectants or the stabilization of proteins during the freeze-drying process.	Excluded
A Comparison between Primary and Secondary Flap Coverage in Ridge Preservation Procedures: A Pilot Randomized Controlled Clinical Trial	2019	https://doi.org/10.1155/2019/7679319	The study does not involve the use of lyoprotectants or freeze-drying processes at all. It is entirely focused on surgical methods and outcomes in dentistry, without addressing the challenges or techniques related to the stabilization of proteins during lyophilization.	Not relevent to topic	Excluded
Safety and immunogenicity of fully liquid and lyophilized formulations of an investigational trivalent group B streptococcus vaccine in healthy non-pregnant women: Results from a randomized comparative phase II trial.	2020	https://doi.org/10.1016/j.vaccine.2020.02.085	vaccine's lyophilized formulation is evaluated, the paper does not explore the technical aspects of lyoprotectants or the mechanisms of protein stabilization during the freeze-drying process.	The research is centered on vaccine formulations rather than the effectiveness of different lyoprotectants in protecting protein structures during the freeze-drying process.	Excluded
The Pharmacokinetics and Relative Bioavailability of Mepolizumab 100 mg Liquid Formulation Administered Subcutaneously to Healthy Participants: A Randomized Trial.	2020	https://doi.org/10.1002/cpdd.726	The study examines a liquid formulation of the drug administered subcutaneously (via injection under the skin), not a freeze-dried version. Since freeze-drying is not involved, there is no exploration of the technical challenges or strategies associated with protein stabilization during lyophilization.	The focus is on the pharmacological behavior of the liquid drug, not on the stabilization of proteins using lyoprotectants.	Excluded
Safety and efficacy of a freeze-dried trivalent antivenom for snakebites in	2017	https://doi.org/10.1371/journal.pntd.0006068	Included because freeze-dried implies that lyoprotectants were used to stabilize the antibodies during the	Inluded	Included

the Brazilian Amazon: An open randomized controlled phase IIb clinical trial.			lyophilization process. The success of the freeze-dried formulation in the clinical trial depends heavily on how well the proteins were stabilized, making the study relevant to the investigation of effective lyoprotectants.		
Pre-marketing immunogenicity and safety of a lyophilized purified human diploid cell rabies vaccine produced from microcarrier cultures: a randomized clinical trial.	2019	https://doi.org/10.1080/21645515.2018.1549450	The focus is on the clinical performance of the lyophilized vaccine rather than the underlying freeze-drying technology or the selection of lyoprotectants.	study does not delve into the scientific details about the lyoprotectants used, their effectiveness, or how they stabilize proteins.	Excluded
A Double-Blind, Placebo-Controlled Trial to Assess Safety and Tolerability of (Thetanix) Bacteroides thetaiotaomicron in Adolescent Crohn's Disease.	2020	https://doi.org/10.14309%2Fctg.0000000000000287	The trial does not address the freeze-drying (lyophilization) process or the stabilization of proteins.	The study's primary aim is to assess the safety and tolerability of Bacteroides thetaiotaomicron in adolescents with Crohn's disease, focusing on its use as a therapeutic probiotic for gut health. This has no direct connection to the use of lyoprotectants or the freeze-drying process for protein stabilization.	Excluded
French lyophilized plasma versus fresh frozen plasma for the initial management of trauma-induced coagulopathy: a randomized open-label trial.	2018	https://doi.org/10.1111/jth.13929	Can be included	study involves lyophilized plasma, it does not discuss the specific role of lyoprotectants or how they stabilize proteins during the freeze-drying process.	Excluded
French lyophilized plasma versus normal saline for post-traumatic coagulopathy prevention and correction: PREHO-PLYO protocol for a multicenter randomized controlled clinical trial.	2020	https://doi.org/10.1186%2Fs13063-020-4049-1	The paper does not investigate the use or effectiveness of lyoprotectants in preserving proteins during the freeze-drying process. While lyophilized plasma is involved, the research does not delve into how plasma proteins are stabilized or protected during the freeze-drying process, which is a key aspect when studying lyoprotectants.	study uses a freeze-dried product, it does not explore how various lyoprotectants contribute to the stability of proteins during lyophilization.	Excluded

Safety and preliminary efficacy of orally administered lyophilized fecal microbiota product compared with frozen product given by enema for recurrent Clostridium difficile infection: A randomized clinical trial.	2018	https://doi.org/10.1371/journal.pone.0205064	The trial does not address the freeze-drying (lyophilization) process or the stabilization of proteins.	Not relevent to topic	Excluded
Immunological non-inferiority of a new fully liquid presentation of the MenACWY-CRM vaccine to the licensed vaccine: results from a randomized, controlled, observer-blind study in adolescents and young adults.	2022	https://doi.org/10.1080/21645515.2021.1981085	The study compares a fully liquid presentation of the MenACWY-CRM vaccine to a licensed version. The emphasis is on ensuring immunological equivalence between two liquid vaccine formulations, with no exploration of freeze-drying processes or lyophilization.	The study does not involve any discussion or testing of lyoprotectants or the effectiveness of protecting proteins during the freeze-drying process, making it unrelated to the specific challenges of protein preservation in lyophilization.	Excluded
Lyophilization and stability of antibody-conjugated mesoporous silica nanoparticle with cationic polymer and PEG for siRNA delivery	Publication Year	http://dx.doi.org/10.2147/IJN.S164393	Relevent to study. Lyoprotectant explored. Effect on protein model discussed	Relevent to study. Lyoprotectant explored. Effect on protein model discussed	Included
Using Some Lyoprotectants for Shelf Life Improvement of a Lyophilized Intravesical Immune	2018	https://eijppr.com/storage/models/article/wS3VlstemUg5FO1ZtdsdLVHj4Xng9V3CnL79m2e19eGf0dEj3LqMqOIFRhbk/using-some-lyoprotectants-for-shelf-life-improvement-of-a-lyophilized-intravesical-immune-bcg.pdf	Relevent to study. Lyoprotectant explored. Effect on protein model discussed	Relevent to study. Lyoprotectant explored. Effect on protein model discussed	Included
Thermostable lyoprotectant-enhanced cell-free protein synthesis for on-demand	2018	http://dx.doi.org/10.1016/j.nbt.2019.07.004	Relevent to study. Lyoprotectant explored. Effect on protein model discussed	Relevent to study. Lyoprotectant explored. Effect on protein model discussed	Included

endotoxin-free therapeutic production					
Lyoprotection and stabilization of laccase extract from Coriolus hirsutus, using selected additives	2019	http://dx.doi.org/10.1186/s13568-018-0683-3	Relevent to study. Lyoprotectant explored. Effect on protein model discussed	Relevent to study. Lyoprotectant explored. Effect on protein model discussed	Included
Designing the Optimal Formulation for Biopharmaceuticals: A New Approach Combining Molecular Dynamics and Experiments	2018	http://dx.doi.org/10.1016/j.xphs.2018.09.002	Relevent to study. Lyoprotectant explored. Effect on protein model discussed	Relevent to study. Lyoprotectant explored. Effect on protein model discussed	Included
Short communication: Global transcriptome analysis of Lactococcus lactis ssp. lactis in response to gradient freezing	2019	http://dx.doi.org/10.3168/jds.2018-15972	The study may not evaluate or identify specific lyoprotectants or their efficacy in protecting proteins during freeze-drying, making it less relevant to practical applications in protein stabilization.	The research may not include specific analyses related to protein stability, activity, or preservation during lyophilization, which are crucial aspects when evaluating lyoprotectants.	Excluded
Screening of novel excipients for freeze-dried protein formulations	2019	https://doi.org/10.1016/j.ejpb.2021.01.008	Relevent to study. Lyoprotectant explored. Effect on protein model discussed	Relevent to study. Lyoprotectant explored. Effect on protein model discussed	Included
Exploring the chemical space for freeze-drying excipients	2021	https://doi.org/10.1016/j.ijpharm.2019.05.065	The wrong type of manuscript. This was an accepted manuscript but not published article.	Relevent to topic but cannot be included due to type of manuscript	Excluded
Lyophilization of premixed COVID-19 diagnostic RT-qPCR reactions enables stable long-term storage at elevated temperature	2019	https://doi.org/10.1002/biot.202000572	Relevent to study. Lyoprotectant explored. Effect on protein model discussed	Relevent to study. Lyoprotectant explored. Effect on protein model discussed	Included
Effects of nanofibrillated cellulose hydrogels on adipose tissue extract and hepatocellular carcinoma cell spheroids in freeze-drying	2021	https://doi.org/10.1016/j.cryobiol.2019.09.005	The study is primarily concerned with the preservation of tissue extracts and cell spheroids, rather than proteins.	The study investigates nanofibrillated cellulose hydrogels, which may function more as structural stabilizers for tissue or cells rather than specific lyoprotectants that target protein stability.	Excluded
Design of a new lyoprotectant increasing freeze-dried	2019	Design of a new lyoprotectant increasing freeze-dried	Relevent to study. Lyoprotectant explored. Effect on protein model discussed	Relevent to study. Lyoprotectant explored. Effect on protein model discussed	Included

Lactobacillus strain survival to long-term storage		Lactobacillus strain survival to long-term storage | BMC Biotechnology			
Stabilization of RNA Encapsulated in Silk	2021	https://pubs.acs.org/doi/10.1021/acsbiomaterials.8b00207	The study may focus more on encapsulation methods and their effects on RNA rather than on lyoprotection during freeze-drying, which is a crucial aspect of effective lyoprotectants for protein formulations.	The study examines silk as a matrix for RNA stabilization. The properties and interactions of silk may not translate to effective lyoprotectants for proteins.	Excluded
Impact of protectants on the storage stability of freeze-dried probiotic Lactobacillus plantarum	2018	http://dx.doi.org/10.3168/jds.2018-15972	f the research does not evaluate how the protectants impact the stability or activity of proteins, it will not provide relevant insights into their effectiveness as lyoprotectants for protein formulations.	The study investigates the stability of freeze-dried Lactobacillus plantarum, a probiotic bacterium, rather than focusing on proteins. The mechanisms of stabilization for live cells differ significantly from those needed for preserving protein structure and function during freeze-drying.	Excluded
Improved viability of Lactobacillus plantarum embedded in whey protein concentrate/pullulan/trehalose hydrogel during freeze drying	2019	https://doi.org/10.1016/j.ejpb.2021.01.009	study does not evaluate the stability, activity, or structural integrity of proteins during the freeze-drying process, it lacks relevance for determining effective lyoprotectants for protein formulations.	If the study draws broad conclusions about the protective effects of the hydrogel system without specific insights into how these findings translate to protein stabilization, the relevance to protein lyoprotection may be limited.	Excluded
Evaluation of total monomeric anthocyanin, total phenolic content and individual anthocyanins of foam-mat freeze-dried and spray-dried blueberry powder	2021	https://doi.org/10.1016/j.ijpharm.2019.05.066	Relevent to study. Lyoprotectant explored. Effect on protein model discussed	Relevent to study. Lyoprotectant explored. Effect on protein model discussed	Included
Surface characteristics and proteomic analysis insights on the response of Oenococcus oeni SD-2a to freeze-drying stress	2019	10.1016/j.foodchem.2018.04.137	Research does not evaluate specific lyoprotectants or their effects on protein stability, it may not provide relevant information for the	tudy draws general conclusions about microbial responses without specific relevance to protein stabilization mechanisms, its findings may not	Excluded

			development of effective protein formulations.	contribute to understanding effective lyoprotectants for protein formulations.	
ANALYSIS OF THE EXISTING METHODS AND SPECIFIC FEATURES OF DRYING SHIITAKE MUSHROOMS		10.15673/fst.v15i3.2118	The research may not assess specific lyoprotectants that are effective for protein formulations, making it less relevant to the quest for optimal protective agents in the freeze-drying process of proteins.	The study concentrates on the drying methods for shiitake mushrooms, which are a type of food product rather than proteins.	Excluded
Nanoencapsulation of lemon essential oil in Chitosan-Hicap system. Part 1: Study on its physical and structural characteristics		10.1016/j.ijbiomac.2018.04.038	The study may not evaluate specific lyoprotectants that enhance protein stability during freeze-drying, focusing instead on the encapsulation of bioactive compounds, making it less applicable to protein stabilization.	The chemical properties of essential oils and proteins vary significantly, leading to different stabilization requirements. The findings from this study may not translate to effective strategies for protein stabilization.	Excluded
QUALITY ASSESSMENT OF EXPERIMENTAL COOKIES ENRICHED WITH FREEZE-DRIED BLACK CHOKEBERRY		https://doi.org/10.17306/j.afs.0686	The challenges involved in preserving the quality of cookies, such as texture, flavor, and nutritional value, differ from the specific stabilization needs of proteins, which involve preventing denaturation, aggregation, and maintaining bioactivity.	The study may not evaluate specific lyoprotectants that are effective for stabilizing proteins during freeze-drying, making it less applicable to the topic of protein stabilization.	Excluded
Improvement of Quality and Digestibility of Moringa Oleifera Leaves Feed via Solid-State Fermentation by Aspergillus Niger		http://dx.doi.org/10.1515/ijcre-2018-0094	The study may not assess specific lyoprotectants or protective agents that can stabilize proteins during freeze-drying, making it less applicable to the topic of protein stabilization.	The study primarily investigates the improvement of feed quality and digestibility of Moringa oleifera leaves through solid-state fermentation, which is distinct from the stabilization of proteins during freeze-drying. The focus on fermentation by Aspergillus niger may highlight microbial interactions and metabolic processes rather than the physical or chemical stabilization needed for proteins in freeze-dried formulations.	Excluded
Comparison of the performance of secretome analysis based on metabolic		https://doi.org/10.3724/sp.j.1123.2021.04017	The study concentrates on secretome analysis, which involves examining the proteins secreted by cells rather	The study may not evaluate specific lyoprotectants or protective strategies that enhance protein stability during	Excluded

labeling by three unnatural sugars			than addressing the stabilization of proteins during freeze-drying. The methodologies and objectives related to secretome analysis differ significantly from those required for effective protein preservation.	freeze-drying, making it less applicable to the topic of protein preservation.	
Impact of protectants on the storage stability of freeze-dried probiotic Lactobacillus plantarum		https://doi.org/10.1007/s10068-018-0523-x	The protectants used in this study are likely aimed at ensuring the survival and activity of probiotics, which involves maintaining cell membrane integrity and metabolic function. In contrast, effective lyoprotectants for proteins must focus on preventing denaturation, aggregation, and loss of biological activity of proteins.	The findings regarding protectants for probiotics may not apply to the preservation of proteins, which have distinct structural and chemical properties.	Excluded
Enzymatic characterization of D-lactate dehydrogenase and application in alanine aminotransferase activity assay kit		https://doi.org/10.1080/21655979.2021.1972781	The study may not evaluate specific lyoprotectants or strategies that enhance the stability of enzymes (or other proteins) during freeze-drying, making it less applicable to the topic of protein stabilization.	The main goal of this research is to characterize the enzymatic activity and potential applications of D-lactate dehydrogenase, which does not directly relate to the challenges of maintaining protein stability during freeze-drying processes.	Excluded
Effect of Lyophilization on Stability of PEG-Protein Conjugate: A Case Study with Peginterferon alfa-2b		http://dx.doi.org/10.1590/s2175-97902023e201120	If the study does not comprehensively evaluate various lyoprotectants or protective strategies in relation to the stability of PEG-protein conjugates during freeze-drying, its relevance to the development of effective lyoprotectants may be limited.	study does not comprehensively evaluate various lyoprotectants or protective strategies in relation to the stability of PEG-protein conjugates during freeze-drying, its relevance to the development of effective lyoprotectants may be limited.	Excluded
A strategy to promote the convenient storage and direct use of polyhydroxybutyrate-degrading Bacillus sp. JY14 by lyophilization with protective reagents	2023	https://doi.org/10.1186/s12934-023-02173-4	Relevent to study. Lyoprotectant explored. Effect on protein model discussed	Relevent to study. Lyoprotectant explored. Effect on protein model discussed	Included

Lyophilization for Formulation Optimization of Drug-Loaded Thermoresponsive Polyelectrolyte Complex Nanogels from Functionalized Hyaluronic Acid	2023	**https://doi.org/10.3390/pharmaceutics15030929**	The study may not explore specific lyoprotectants or protective agents that enhance protein stability during freeze-drying, making it less applicable to the topic of effective protein stabilization.	Findings related to the stabilization of drug-loaded nanogels may not be directly applicable to the stabilization of proteins, as different factors influence the stability and activity of drugs and proteins during lyophilization.	Excluded
Calcium ions enhanced freeze-drying and spray-drying resistance of Lactiplantibacillus plantarum LIP-1 by regulation of cell wall surface proteins and related enzymes expression	2023	https://doi.org/10.1016/j.lwt.2023.115460	Relevent to study. Lyoprotectant explored. Effect on protein model discussed	Relevent to study. Lyoprotectant explored. Effect on protein model discussed	Included
Stabilising and functional effects of Spirulina (Arthrospira platensis) protein isolate on encapsulated Lacticaseibacillus rhamnosus GG during processing, storage and gastrointestinal digestion	2023	https://doi.org/10.1016/j.foodhyd.2023.109519	Relevent to study. Lyoprotectant explored. Effect on protein model discussed	Relevent to study. Lyoprotectant explored. Effect on protein model discussed	Included
In-situ investigation of solid phase evolution during lyophilization of mannitol-based antibody formulations using an XRPD climate chamber	2023	10.1016/j.ejps.2023.106407	the study does not evaluate the role of lyoprotectants in stabilizing antibodies during the lyophilization process, its relevance to the broader discussion of effective lyoprotectants for protein formulations may be limited.	the research does not provide a thorough examination of how the solid-state changes impact the stability or activity of the antibody formulations, it may not contribute meaningfully to understanding effective strategies for lyoprotection.	Excluded
Lyophilization process optimization and molecular dynamics simulation of mRNA-LNPs for SARS-CoV-2 vaccine	2023	https://doi.org/10.1038/s41541-023-00732-9	Relevent to study. Lyoprotectant explored. Effect on protein model discussed	Relevent to study. Lyoprotectant explored. Effect on protein model discussed	Included
Physicochemical, Structural, and Functional Properties of	2023	https://doi.org/10.3390/foods12142679	The study primarily investigates the microencapsulation of snake melon	The objectives of this research are likely centered on improving the	Excluded

Snake Melon (Cucumis melo subsp. melo Var. flexuosus) Microencapsulated with Pea Protein and Pea Fibre by Freeze-Drying			using pea protein and fiber, which focuses on the encapsulation process rather than the stabilization of free proteins.	physicochemical and functional properties of the encapsulated product, rather than addressing the challenges associated with maintaining protein stability during freeze-drying.	
Enhanced Stability of Vegetal Diamine Oxidase with Trehalose and Sucrose as Cryoprotectants: Mechanistic Insights	2023	https://doi.org/10.3390/molecules28030992	Relevent to study. Lyoprotectant explored. Effect on protein model discussed	Relevent to study. Lyoprotectant explored. Effect on protein model discussed	Included
Maltodextrin as a Drying Adjuvant in the Lyophilization of Tropical Red Fruit Blend	2023	https://doi.org/10.3390/molecules28186596	The study primarily investigates the use of maltodextrin as a drying adjuvant in the lyophilization of tropical red fruit blends. The focus on fruit composition may not provide insights relevant to the stabilization of proteins, which have distinct biochemical properties and stabilization requirements.	The behavior of maltodextrin as a carbohydrate may not correlate with the mechanisms required to stabilize proteins, as different types of compounds interact differently with proteins during freeze-drying.	Excluded
Lyoprotectant Formulation and Optimization of the J-Aggregates Astaxanthin/BSA/Chitosan Nanosuspension	2023	https://doi.org/10.3390/biom13030496	he study may not explore the role of lyoprotectants in a broader context beyond the specific formulation of astaxanthin/BSA/chitosan, which may limit its applicability to other protein formulations that require lyoprotection.	the research does not thoroughly evaluate how the lyoprotectants impact the stability or functionality of BSA or other proteins in the context of freeze-drying, its relevance to effective lyoprotection may be limited.	Excluded
The Effect of Decreased Ca++/Mg++ ATPase Activity on Lactobacillus delbrueckii subsp. bulgaricus sp1.1 Survival during Spray Drying	2023	https://doi.org/10.3390/foods12040787	The research focuses on spray drying, which operates under different conditions compared to freeze-drying (lyophilization).	research may not specifically assess lyoprotectants that are effective in preserving protein stability during freeze-drying, limiting its applicability to the topic of effective protein stabilization.	Excluded
Effect of Lyoprotective Agents on the Preservation of Survival of a Bacillus cereus Strain PBG in the Freeze-Drying Process	2023	https://doi.org/10.3390/microorganisms11112705	Relevent to study. Lyoprotectant explored. Effect on protein model discussed	Relevent to study. Lyoprotectant explored. Effect on protein model discussed	Included

Clearance of Trehalose Lyophilized Platelets in Mice	2023	http://dx.doi.org/10.2478/acve-2023-0039	The study primarily investigates the clearance of trehalose lyophilized platelets in a biological model. This focus on the clearance and behavior of a specific cell type may not provide relevant insights into the stabilization of proteins during freeze-drying, as the mechanisms and requirements for cellular stability differ from those needed for isolated proteins.	Not relevent to topic	Excluded
Leuconostoc performance in soy-based fermentations-Survival, acidification, sugar metabolism, and flavor comparisons	2023	https://doi.org/10.1016/j.fm.2023.104337	study may not specifically investigate the role of lyoprotectants in stabilizing proteins during the fermentation and subsequent processing stages, limiting its applicability to the topic of effective protein stabilization during freeze-drying.	The findings related to soy-based fermentation and flavor profiles may not generalize to other protein sources or formulations, making them less applicable to broader discussions about protein stabilization during lyophilization.	Excluded
Application of Quality by Design Principles to the Development of Oral Lyophilizates Containing Olanzapine	2023	https://doi.org/10.3390/pharmaceutics15071967	research does not examine the effects of the formulation on protein stability or incorporate proteins as part of the lyophilizate, it may not provide relevant insights into effective lyoprotection strategies for proteins.	not focus on identifying or evaluating lyoprotectants specifically aimed at improving protein stabilization during freeze-drying, limiting its relevance to the topic.	Excluded
Freeze-Drying of Encapsulated Bacteriophage T4 to Obtain Shelf-Stable Dry Preparations for Oral Application	2023	https://doi.org/10.3390/pharmaceutics15122792	not specifically address lyoprotectants that are effective for stabilizing proteins	findings related to the freeze-drying of encapsulated bacteriophage T4 may not generalize to other protein formulations, making them less applicable to broader discussions about protein stabilization during lyophilization.	Excluded
Screening and Stability Evaluation of Freeze-Dried Protective Agents for a Live Recombinant Pseudorabies Virus Vaccine	2023	https://doi.org/10.3390/vaccines12010065	Relevent to study. Lyoprotectant explored. Effect on protein model discussed	Relevent to study. Lyoprotectant explored. Effect on protein model discussed	Included
Optimized biomimetic minerals maintain activity of	2023	https://doi.org/10.1016/j.actbio.2023.11.044	research does not examine the effects of storage conditions on the stability	not specifically evaluate lyoprotectants that are effective in preserving proteins	Excluded

mRNA complexes after long term storage			or activity of proteins in conjunction with mRNA, it may not provide relevant insights into effective lyoprotection strategies specifically for proteins.	during freeze-drying, limiting its applicability to the topic of effective protein stabilization.	
Lysozyme-Sucrose Interactions in the Solid State: Glass Transition, Denaturation, and the Effect of Residual Water	2023	https://doi.org/10.1021/acs.molpharmaceut.3c00403	research does not comprehensively assess how the lysozyme-sucrose interactions impact the functional integrity of lysozyme post-lyophilization, it may not provide relevant insights into effective protein stabilization strategies during freeze-drying.	Not relevent to topic	Excluded
New insights into the protein stabilizing effects of trehalose by comparing with sucrose	2023	https://doi.org/10.1039/D3CP02639F	Relevent to study. Lyoprotectant explored. Effect on protein model discussed	Relevent to study. Lyoprotectant explored. Effect on protein model discussed	Included
Ranking mAb-excipient interactions in biologics formulations by NMR spectroscopy and computational approaches	2023	https://doi.org/10.1080/19420862.2023.2212416	he study centers on the interactions between monoclonal antibodies (mAbs) and excipients, which may not directly address the specific mechanisms and challenges associated with lyoprotection during freeze-drying.	research does not specifically evaluate the effectiveness of the excipients in stabilizing mAbs during freeze-drying, its relevance to the development of effective lyoprotectants for protein stabilization may be limited.	Excluded
How Sugars Protect Dry Protein Structure	2023	https://doi.org/10.1021%2Facs.biochem.2c00692	not specifically address lyoprotectants that are effective for stabilizing proteins	not specifically evaluate lyoprotectants that are effective in preserving proteins during freeze-drying.	Excluded
Accelerated stability testing and simulated gastrointestinal release of encapsulated betacyanins and phenolic compounds from Bougainvillea glabra bracts extract	2022	https://www.scopus.com/inward/record.uri?eid=2-s2.0-85131691230&doi=10.1016%2fj.foodchem.2022.133391&partnerID=40&md	not specifically address the role of lyoprotectants in stabilizing proteins during freeze-drying	study centers on the stability and gastrointestinal release of encapsulated phytochemicals (betacyanins and phenolic compounds) from a plant extract, rather than on the stabilization of proteins. The mechanisms that govern the stability of these compounds can differ significantly from those required for protein stabilization during freeze-drying.	Excluded
Development of (Inhalable) Dry Powder Formulations of	2022	https://www.scopus.com/inward/record.uri?ei	research does not focus specifically on the stability of proteins (such as	stabilization mechanisms for vaccine formulations, especially those	Excluded

AS01B-Containing Vaccines Using Thin-Film Freeze-Drying		d=2-s2.0-85130805981&doi=10.1016%2fj.ijpharm.2022.121825&partnerID=40&md5	antigens) within the vaccine formulation during the freeze-drying	involving adjuvants like AS01B, may differ from those needed for standalone proteins.	
Cryoprotective agents influence viral dosage and thermal stability of inhalable dry powder vaccines	2022	https://www.scopus.com/inward/record.uri?eid=2-s2.0-85125126517&doi=10.1016%2fj.ijpharm.2022.121602&partnerID=40&md5	study does not specifically assess how cryoprotective agents function in stabilizing proteins or their mechanisms during the freeze-drying process	findings may be highly specific to the formulation and stability of inhalable dry powder vaccines, which may not generalize to other types of protein formulations or biologics that undergo freeze-drying.	Excluded
Polyphosphates as an effective vehicle for delivery of bioavailable nanoparticulate iron(III)	2022	https://www.scopus.com/inward/record.uri?eid=2-s2.0-85122511369&doi=10.1016%2fj.foodchem.2021.131477&partnerID=40&md	The study centers on the delivery of nanoparticulate iron(III) using polyphosphates, which pertains to the field of iron supplementation or nutrition rather than protein stabilization.	study does not specifically address the use of polyphosphates as lyoprotectants for proteins or their effectiveness in protecting protein structures during freeze-drying	Excluded
Enhanced stability of stilbene-glycoside-loaded nanoparticles coated with carboxymethyl chitosan and chitosan hydrochloride	2022	https://www.scopus.com/inward/record.uri?eid=2-s2.0-85117127458&doi=10.1016%2fj.foodchem.2021.131343&partnerID=40&md5	study investigates the stability of stilbene-glycoside-loaded nanoparticles, which are different from protein formulations. The stabilization strategies for nanoparticles may not be applicable to proteins, as they often have unique structural and functional characteristics	Research does not evaluate how the stabilization techniques affect proteins (e.g., through co-encapsulation or interactions with the nanoparticles), it may not provide insights directly relevant to effective lyoprotection for proteins.	Excluded
Interaction of ovalbumin with lutein dipalmitate and their effects on the color stability of marigold lutein esters extracts	2022	https://www.scopus.com/inward/record.uri?eid=2-s2.0-85115993626&doi=10.1016%2fj.foodchem.2021.131211&partnerID=40&	The study primarily investigates the interaction between ovalbumin and lutein dipalmitate in the context of color stability rather than protein stabilization during freeze-drying. While color stability can be an important parameter, it may not address the structural integrity and functional activity of proteins, which are critical during freeze-drying.	study does not evaluate lutein dipalmitate or ovalbumin as potential lyoprotectants during freeze-drying processes, its relevance to the topic of lyoprotectants may be limited.	Excluded

Agro-industrial by-products: Valuable sources of bioactive compounds	2022	https://www.scopus.com/inward/record.uri?eid=2-s2.0-85121901353&doi=10.1016%2fj.foodres.2021.110871&partnerID=40&md5	The study centers on the extraction and characterization of bioactive compounds from agro-industrial by-products, which may not specifically address protein stabilization.	study does not specifically assess how the bioactive compounds or by-products can serve as lyoprotectants for proteins during freeze-drying, its relevance to the topic of effective lyoprotectants for protein stabilization	Excluded
A comprehensive review on innovative and advanced stabilization approaches of anthocyanin by modifying structure and controlling environmental factors	2021	https://www.scopus.com/inward/record.uri?eid=2-s2.0-85112330474&doi=10.1016%2fj.foodchem.2021.130611&partnerID=40&md	Excluded because of this is a review.	Excluded because of this is a review.	Excluded
Construction and characterization of antioxidative ferulic acid-grafted carboxylic curdlan conjugates and their contributions on β-carotene storage stability	2021	https://www.scopus.com/inward/record.uri?eid=2-s2.0-85100392070&doi=10.1016%2fj.foodchem.2021.129166&partnerID=40&md	Research does not explore how these conjugates can stabilize proteins or if they do not evaluate the interactions with proteins	study primarily investigates the stability of β-carotene in the presence of ferulic acid-grafted curdlan conjugates. This focus on carotenoid stability does not directly address the unique stabilization challenges and strategies needed for proteins during freeze-drying.	Excluded
Risk assessment of coffees of different qualities and degrees of roasting	2021	https://www.scopus.com/inward/record.uri?eid=2-s2.0-85099478683&doi=10.1016%2fj.foodres.2020.110089&partnerID=40&md5	The study primarily examines the risks associated with different qualities and roasting levels of coffee, which may include aspects such as chemical composition, safety, and consumer health. This focus does not pertain to the stabilization of proteins during freeze-drying.	findings related to coffee quality and safety may not generalize to other types of protein formulations or biologics that require stabilization during freeze-drying, as the mechanisms of stabilization and degradation can differ widely between food products and biological material	Excluded
Cryoprotective effect of antifreeze glycopeptide analogues obtained by nonenzymatic glycation on Streptococcus thermophilus and its possible action mechanism	2019	https://www.scopus.com/inward/record.uri?eid=2-s2.0-85062730517&doi=10.1016%2fj.foodchem.2019.03.011&partnerID=40&md5	Relevent to study. Lyoprotectant explored. Effect on protein model discussed	Relevent to study. Lyoprotectant explored. Effect on protein model discussed	Included

Stabilization of bovine lactoperoxidase in the presence of ectoine	2018	https://www.scopus.com/inward/record.uri?eid=2-s2.0-85047452165&doi=10.1016%2fj.foodchem.2018.05.067&partnerID=40&md5	The stabilization mechanisms observed for lactoperoxidase in the presence of ectoine may differ from those necessary for proteins during the freeze-drying process. The conditions and parameters studied might not replicate the specific stresses encountered during lyophilization.	research focuses solely on lactoperoxidase, it may not provide insights applicable to the stabilization of other proteins, enzymes, or biologics, limiting its relevance to the broader field of lyoprotection	Excluded
Freeze-drying of monoclonal antibody-conjugated gold nanorods: Colloidal stability and biological activity	2018	https://www.scopus.com/inward/record.uri?eid=2-s2.0-85052466985&doi=10.1016%2fj.ijpharm.2018.08.045&partnerID=40&md5	study primarily investigates the stabilization of gold nanorods conjugated with monoclonal antibodies rather than the stabilization of proteins in a more general sense. The interactions and stabilization mechanisms for nanoparticles are different from those required for protein stabilization.	study does not evaluate the effectiveness of ectoine as a lyoprotectant specifically during the freeze-drying process or its impact on protein stability post-lyophilization, its relevance to protein stabilization may be limited.	Excluded
Manufacturing and ambient stability of shelf freeze dried bacteriophage powder formulations	2018	https://www.scopus.com/inward/record.uri?eid=2-s2.0-85042878963&doi=10.1016%2fj.ijpharm.2018.02.023&partnerID=40&md5	Relevent to study. Lyoprotectant explored. Effect on protein model discussed	Relevent to study. Lyoprotectant explored. Effect on protein model discussed	Included

Supplementary Table S2: Preferred Reporting Items for Systematic reviews and Meta-Analyses extension for Scoping Reviews (PRISMA-ScR) Checklist

SECTION	ITEM	PRISMA-ScR CHECKLIST ITEM	REPORTED ON PAGE #
**TITLE**			
Title	1	Effectiveness of lyoprotectants in protein stabilisation during lyophilization.	Page 1
**ABSTRACT**			
Structured summary	2	The abstract provides a clear structured summary on lyoprotectants in protein stabilisation during lyophilization with background, objectives, methods, results, and conclusions.	Page 1
**INTRODUCTION**			
Rationale	3	The introduction outlines the necessity of lyoprotectant during freeze-drying, importance of identifying correct lyoprotectant for protein stabilization.	Page 1-2
Objectives	4	The objectives are clearly stated, focusing on evaluating potential lyoprotectant, their effectiveness of lyoprotectants in protein stabilisation during lyophilization.	Page 2
**METHODS**			
Protocol and registration	5	Not Applicable	NA
Eligibility criteria	6	The criteria are well defined, focusing on original research articles effectiveness of lyoprotectants in protein stabilisation during lyophilization.	Page 3-4

SECTION	ITEM	PRISMA-ScR CHECKLIST ITEM	REPORTED ON PAGE #
Information sources*	7	Comprehensive searches were conducted in PubMed, Scopus, and Web of Science databases, covering literature from 2018 to 2024.	Page 2
Search	8	The search strategy is detailed, including specific keywords and Boolean operators.	Page 2
Selection of sources of evidence	9	The selection process is described, with independent screening by two reviewers and resolution of disagreements by a third reviewer if necessary.	Page 3
Data charting process	10	Data extraction was performed and verified by a second reviewer to ensure accuracy and alignment with the research questions.	Page 3
Data items	11	The data extracted included publication details, types of lyoprotectant, protein model.	Page 3-11
Critical appraisal of individual sources of evidence§	12	If done, provide a rationale for conducting a critical appraisal of included sources of evidence; describe the methods used and how this information was used in any data synthesis (if appropriate).	Not applicable
Synthesis of results	13	No new synthesis of results, focusing on qualitative synthesis and thematic analysis of the included studies.	Page 3-4
**RESULTS AND DISCUSSION**			
Selection of sources of evidence	14	A PRISMA flow diagram is provided to illustrate the selection process, including the number of records identified, screened, and excluded.	Page 3
Characteristics of sources of evidence	15	Characteristics of the included studies, and specific outcomes, are summarized.	Page 4-16
Critical appraisal within sources of evidence	16	If done, present data on critical appraisal of included sources of evidence (see item 12).	Not applicable

SECTION	ITEM	PRISMA-ScR CHECKLIST ITEM	REPORTED ON PAGE #
Results of individual sources of evidence	17	Data from individual sources are summarized in tables and described narratively, focusing on classification of lyoprotectants and its influence on the protein stability	Page 4-16
Synthesis of results	18	The synthesis highlights the variation in study designs, materials, and outcomes, emphasizing the need for standardization and further research.	Page 2-16
Limitations	19	The limitations of the review are discussed, including the small number of studies, variability in methods, and exclusion of non-English articles.	Page 15-16
Conclusions	20	The conclusion provides a clear interpretation of the results, highlighting the potential lyoprotectant for protein stabilization during freeze-drying and suggesting areas for future research.	Page 16
**FUNDING**			
Funding	21	Funding sources are acknowledged, noting that This study was funded through the UKM Research Grant GGPM-2021-022.	Page 16

From: Tricco AC, Lillie E, Zarin W, O'Brien KK, Colquhoun H, Levac D, et al. PRISMA Extension for Scoping Reviews (PRISMAScR): Checklist and Explanation. Ann Intern Med. 2018; 169:467–473. doi: 10.7326/M18-0850.
